# Learning From People With Dementia What Works Well for Well-Being: Interviews and Focus Groups

**DOI:** 10.1093/geront/gnae077

**Published:** 2024-07-02

**Authors:** Noortje Kloos, Annemiek Bielderman, Debby L Gerritsen

**Affiliations:** Department of Primary and Community Care, Radboud Institute for Health Sciences, Radboud Alzheimer Center, University Knowledge Network for Older Adult Care Nijmegen (UKON), Radboud University Medical Center, Nijmegen, Gelderland, The Netherlands; Centre for eHealth and Well-Being Research, Department of Psychology, Health and Technology, University of Twente, Enschede, Overijssel, The Netherlands; Department of Primary and Community Care, Radboud Institute for Health Sciences, Radboud Alzheimer Center, University Knowledge Network for Older Adult Care Nijmegen (UKON), Radboud University Medical Center, Nijmegen, Gelderland, The Netherlands; Department of Primary and Community Care, Radboud Institute for Health Sciences, Radboud Alzheimer Center, University Knowledge Network for Older Adult Care Nijmegen (UKON), Radboud University Medical Center, Nijmegen, Gelderland, The Netherlands

**Keywords:** Enjoyment, Life satisfaction, Qualitative, Universal models

## Abstract

**Background and Objectives:**

Previous research has tended to prioritize the condition of dementia when investigating positive lived experiences, while there is no evidence that well-being becomes fundamentally different when living with dementia. The current exploratory qualitative study examined how people living with dementia describe how they realize their well-being, without treating dementia as a central concern, and specifically addressed people who are successful in maintaining their well-being.

**Research Design and Methods:**

Semistructured face-to-face interviews (*n* = 16) and 2 focus groups (*n* = 13) were conducted with community-dwelling older people living with dementia, aged 65–93 years (68% male). Conversations covered contributors to experienced life satisfaction, and life enjoyment, and were analyzed using inductive thematic analysis.

**Results:**

Two main themes described how people realize well-being. (1) *To live a fulfilling life*, participants engaged in activities in order to feel useful and relaxed, and they engaged with others, by interacting and sharing with others, and relying on others. (2) *To have a positive attitude toward life*, participants appreciated the good things in their current life, their lived life, and about their own person, and positively coped with difficulties by accepting them as part of life, not dwelling on the negative, and actively addressing difficulties.

**Discussion and Implications:**

The results appear to reflect universal ways of realizing well-being, justifying the use of universal models of well-being for people living with dementia. We can learn from people living with dementia that living a fulfilling life and having a positive attitude toward life are key to realizing their well-being.

## Background and Objectives

While living with dementia used to be mainly described in terms of challenges and losses (Steeman, 2006), it is increasingly recognized that people continue to have positive experiences when having dementia (Wolverson et al., 2016). Living well with dementia is therefore increasingly being studied, but the central starting point in such research remains living life *with dementia*, thus emphasizing the condition (Gerritsen, 2021). The current study aims to investigate how people living with dementia describe *living a good life*, without treating dementia as a central concern.

What it means to be living a good life has been a topic of much debate, and positive psychology offers a useful framework of understanding well-being (Rusk & Waters, 2013). Well-being can be described in terms of feeling well: experiencing positive emotions, few negative emotions, and feeling satisfied with life, which is captured in the concept of subjective well-being (Diener et al., 1985). Well-being is also described as fulfilling one’s potential: experiencing psychological growth, self-acceptance, reaching goals in life (psychological well-being), and feeling part of society (social well-being; Keyes, 1998; Ryff, 1989). Striving for psychological and social well-being may lead to a stable experience of subjective well-being (Deci & Ryan, 2008). Examining how people realize subjective well-being may therefore also address psychological and social well-being.

Even though well-being is a very subjective experience, research in the context of dementia has often relied on proxy or informant assessments (Heuer & Willer, 2020). The validity of such proxy accounts has been questioned, as it proves difficult to assess the well-being of others (Kloos et al., 2020), and people without dementia tend to assess life with dementia more negatively than people living with dementia (Römhild et al., 2018). In terms of ethics, as people with dementia are often able to communicate their personal experiences of living a good life, it could be considered their right to have their voice heard (Cohen-Mansfield, 2021). Indeed, efforts are invested in including self-reports of people with dementia as much as possible in recent research (Perfect et al., 2021).

A recent scoping review of reviews has taken important steps toward conceptualizing a framework of living well with dementia based on self-reports (Clarke et al., 2020). This framework included six aspects: feeling well; feeling positive; keeping going and being active; having good relationships; having a positive sense of self; and one’s life having meaning. However, this work did not take into account that most studies thus far have tended to highlight the condition of dementia as a central concern. For instance, included studies investigated adjustment and coping with dementia (e.g., Hulko, 2009; Wolverson, 2010), or how the lived life has changed because of dementia (e.g., Harris, 2006). Other studies included dementia-specific questionnaires preceding the interview (thereby potentially influencing the following answers; e.g., Byrne-Davis, 2006; Siblerfeld, 2002), or used a dementia-specific framework to analyze the data (Dröes et al., 2006). It is important to consider the potential effects of these aspects during conversations or analytical approach.

Treating dementia as a central concern when examining well-being presumes that living a good life becomes fundamentally different when one is living with dementia. However, this has no evidence in literature, and may result in overemphasizing the aspects of life that are relevant to the dementia, while disregarding aspects that are not affected by the condition though still important for experiencing well-being (Gerritsen, 2021). Importantly, providing person-centered care includes shifting the focus from the condition and related limitations toward the individual and their personhood (Kitwood, 1997). Viewing people with dementia as fundamentally different from ourselves may also strengthen negative stigma (Moyle, 2011), and limits our opportunity to learn from how persons with dementia are able to realize well-being while having a degenerative condition. It is thus important to explore what it means to be living a good life when living with dementia, without differentiating a person’s experience based on their condition in advance.

The current qualitative study takes an exploratory approach and aims to examine how people with early dementia realize their well-being. Their perspective is taken as a directive, without making dementia the central focus in the conversations or analytical approach. Furthermore, we aim to specifically address people who are successful in maintaining their well-being, to shed light on the positive aspects of living with dementia, and to learn from them what works well and to inform future interventions.

## Research Design and Methods

### Study Design

This qualitative study combined 16 individual face-to-face interviews with two face-to-face focus group discussions (*n* = 13; 5 and 8 persons per group, respectively) with people living with dementia, between April and July 2021 in the Netherlands. We tried to achieve not treating dementia as a central concern by asking participants to merely describe their well-being experience (i.e., not *despite* or *while coping with* dementia), without initiating the topic of dementia before or during the conversations, and without using any dementia-specific framework for analysis. We used a semistructured topic guide, and adopted inductive thematic analysis with data saturation as guiding principle. This study adhered to the consolidated criteria for reporting qualitative research and Standards for Reporting Qualitative Research (O’Brien et al., 2014; Tong et al., 2007).

### Recruitment and Sample

Participants were recruited using convenience sampling through contact persons: managers of dementia day care facilities (*n* = 24 participants), and dementia case managers (who arrange care for community-dwelling people with dementia; *n* = 5 participants). This sampling method was chosen, as it offered a safe context for potential participants for first contact, and because this is a hard-to-reach population. We aimed to include people living with dementia who experienced high levels of well-being, with the recruitment message “Well-being in people with memory problems or (early stage) dementia: do you have a positive outlook on life and would you like to share your experiences with us?” Contact persons assessed their clients on inclusion criteria: having dementia, high self-reported well-being, living at home, and able to communicate about experiences. Exclusion criteria were: severe dementia, and dementia due to human immunodeficiency virus (HIV), traumatic brain damage, Down syndrome, or Huntington’s disease. The contact persons informed potential participants about the study and provided an information package. Participants signed up for the study via their contact person, or by contacting the researchers. This recruitment procedure did not allow gathering information concerning reasons for refusal to participate.

### Data Collection

All interviews were facilitated by the first author (N. Kloos, PhD in psychology and experience with interviewing older adults). The focus group discussions were facilitated by N. Kloos and an observer (Charlotte van Corven, dementia researcher, and experienced observer), both female and with no prior relationship with the participants. The conversations took place at the participants’ home (12 interviews), or their day care facility (four interviews; both focus groups). Solely the researchers and persons living with dementia were present, except for four interviews where an informal caregiver was present but not involved in the interview (*n* = 3), or facilitated the participant in answering the questions, per the request of the participant (*n* = 1; including only the responses of the participant in the analyses). No repeat interviews were carried out.

All interviews were conducted and analyzed in Dutch. The interviewer explained the study’s aim and procedure and answered any remaining questions. In accordance with the Dutch Central Committee on Research Involving Human Subjects regulations, the subsequent written informed consent procedure included reassuring participants they could cease participation at any moment. A brief sociodemographic questionnaire (e.g., age, living situation, subjective health) was administered to the participants, whenever possible. Additional sociodemographic information, including dementia diagnosis, was gathered from informal caregivers and/or contact persons *after* concluding the interviews. Interview durations were 18–64 min (*M* = 42 min) and focus group durations were 45 and 60 min. The conversations were audio-recorded and transcribed verbatim by independent researchers; transcripts were not returned to participants.

### Interview and Focus Group Discussion Guides

The topic guides explored experienced well-being by people living with dementia (see Supplementary Material). The topic of memory problems or dementia itself was distinctly *not* part of the guides. As well-being is a rather abstract concept, we opted for more concrete questions, asking to indicate to what extent they experienced life satisfaction and life enjoyment. These represent the cognitive–evaluative and affective components of subjective well-being (Diener et al., 1999). Furthermore, all participants were asked to indicate this on a Visual Analogue Scale from 1 to 10, to offer a visual aid and help orient participants toward accessing their experience of well-being, a starting point for the subsequent open-ended questions that asked about important contributors to well-being (e.g., “What comes to mind when you think about enjoying life?, What makes this an enjoyable day?, Do you have to try your best to make the most of the day?, What would you advise others for enjoying their life?”), including follow-up questions on the topics introduced by the participant(s) (e.g., “What do you mean exactly?, Could you give an example?, Why is that important to you?”).

Interview and discussion guides evolved during data gathering, to gain more insight into the topics introduced by participants. Following each interview and focus group, N. Kloos used field notes to debrief the other research team members about the main content. Subsequent research team discussions informed both the issue of saturation, and adaptations to the conversation guides in an iterative process. The question “What do you have to do to have a positive outlook on life?” was discussed as the main question in both focus groups, and added to each subsequent interview. The final four interviews additionally asked participants to provide feedback on five topics discussed in previous interviews (i.e., *appreciating the good things in life*, *not dwelling on difficulties*, *appreciating one’s lived life*, *doing what you still can*, *helping others*).

### Ethical Considerations

The study was not subject to the Medical Research Involving Human Subjects Act, as declared by the local Ethics Review Committee after reviewing the protocol (no. 2019-5857), and was conducted in accordance with Dutch law and the Code of Conduct Health Research (Coreon, 2022), and the Declaration of Helsinki.

### Data Analysis

The transcripts of the interviews and focus groups were analyzed together, using Atlas.ti (version 9.0). This study used inductive thematic analysis as described by Braun and Clarke (2006) with constant comparison (Green & Thorogood, 2014), and a focus on cross-case analysis. No theoretical framework was used for this analysis, and themes were derived from the data using open coding. Two researchers (Anita Oude Bos, N. Kloos) independently analyzed transcripts of the first five interviews. They compared and discussed codes until consensus was reached, to create a coding system of open codes covering all aspects related to well-being described by the participants (Hsieh & Shannon, 2005). The following 11 interviews and two focus groups were then coded with the coding system by one researcher (AOB), who generated new codes when new topics arose. All resulting codes were then discussed with N. Kloos and a third researcher (A. Bielderman) until consensus was reached, resulting in merging and subdividing some codes. Codes were grouped into categories and higher-order themes by N. Kloos and A. Bielderman in consultation with two additional researchers (D. Gerritsen, Mandy Wijnen), using sticky notes as well as the digital program Miro. For example, the codes “helping others” and “keeping the household running” were grouped into the category of *feeling useful*. No distinction was made between the well-being components of satisfaction and enjoyment in the description of themes, subthemes, and categories. This was in line with the indivisibility in the narrative of the participants. Quotations used in this paper were literally translated in English, with culturally appropriate pseudonyms; identifiable details were removed.

## Results

### Participants

Demographic characteristics of the participants (*n* = 29) are provided in Table 1. Interview participants (*n* = 16) were comparable to focus group participants (*n* = 13). Participants had a mean age of 80 years (range 65–93 years), with 48% aged 65–79 years. Most participants were male, lived independently with a partner, had children, and 59% reported being in *good*, *very good*, or *excellent* subjective health (not in table). All except one focus group participant (who was Curaçaoan) were White and Dutch.

**Table 1.** Demographic Characteristics of Participants in the Interviews, Focus Groups, and Combined Total Group

Sample characteristics	Interview (*n* = 16)	Focus group (*n* = 13)	Total group (*n* = 29)
Age, *M* (*SD*)	78.0 (6.3)	82.7 (5.6)	80.1 (6.4)
Sex, *n* (%)			
Female	3 (19)	1 (8)	4 (14)
Male	13 (81)	12 (92)	25 (86)
Living situation, *n* (%)			
Independently with partner	9 (56)	13 (100)	22 (76)
Independently alone	7 (44)	—	7 (44)
Children, *n* (%)			
Yes	14 (88)	12 (92)	26 (90)
No	2 (13)	1 (8)	3 (10)
Education, *n* (%)^a^			
Low	7 (44)	—	—
Intermediate	3 (19)	—	—
High	4 (25)	—	—
Unknown	2 (13)	—	—
Memory problems stage, *n* (%)^b^			
Early (mild)	1 (6)	5 (39)	6 (21)
Medium (moderate)	8 (50)	3 (23)	11 (38)
Late (severe)	—	—	—
Unknown	7 (44)	5 (39)	12 (41)
Dementia diagnosis, *n* (%)			
Vascular dementia	3 (19)	2 (15)	5 (17)
Alzheimer’s disease	2 (13)	—	2 (7)
Dementia by Parkinson’s disease	1 (6)	1 (8)	2 (7)
Dementia by Lewy bodies	1 (6)	—	1 (3)
Alcohol dementia	—	1 (8)	1 (3)
Mixed	1 (6)	1 (8)	2 (7)
Unknown/other	8 (50)	8 (62)	16 (55)

*Notes*: *SD* = standard deviation.

^a^Education level was not assessed for focus group participants, due to time restraints.

^b^As assessed by informal caregivers and contact persons.

Most participants described a good level of life satisfaction (*M* = 7.5, standard deviation [*SD*] = 1.3) and life enjoyment (*M* = 7.5, *SD* = 1.0, not in table), although four participants reported a score of 4–6 on one or both outcomes, indicating that they did not experience high well-being.

### Main Themes

The analysis resulted in two themes as important ways for people with dementia to experience well-being: (1) *living a fulfilling life* and (2) *having a positive attitude toward life*. Both main themes were addressed in each interview and both focus groups. The last few interviews did not result in any new themes or subthemes, indicating that data saturation was reached. The interview where the informal caregiver facilitated the participant answers provided fewer and less rich data; no other apparent differences were observed in the interviews where the informal caregiver was present: these participants did not appear to talk less freely, nor did their description of the informal caregiver’s influence on their well-being seem different from the other interviews.

Dementia was spontaneously mentioned by participants in 15 of 16 interviews and both focus group discussions, at varying stages of the interview. Several participants described how they got their diagnosis and their initial reaction to it (e.g., anxiety because of previous experience with others with dementia). The degenerative nature of the condition was also mentioned; however, participants seemed to be mostly at peace with their current condition. Memory problems were mentioned in the context of subjective health valuation by a few participants. The mentions of dementia related to the well-being themes are described below. The main themes, subthemes, and categories are listed in Table 2.

**Table 2.** Themes, Subthemes, and Categories From the Combined Data of Interviews and Focus Groups

Main theme	Subtheme	Category
Living a fulfilling life	Engaging in activities	Feeling useful
		Relaxing
	Engaging with others	Interacting with others
		Sharing with others
		Relying on others
Having a positive attitude toward life	Appreciating the good things in life	Appreciating current life
		Appreciating the lived life
		Appreciating oneself
	Coping with difficulties in a positive way	Accepting difficulties as being part of life
		Not dwelling on difficulties
		Actively addressing difficulties

### Theme “Living a Fulfilling Life”

One of the identified two main themes of how to realize well-being was living a fulfilling life. This theme describes how people ensure that the content of their current life is meaningful, both in terms of *engaging in activities* and *engaging with others*, which was addressed in all interviews except one, and both focus groups.

#### Subtheme: engaging in activities

Engaging in enough meaningful and fun activities was important to keep busy, and a day care facility supported some participants in engaging in activities. Participants felt the freedom to make their own choices of what activities to pursue, and emphasized the importance of using their strengths and abilities in the current moment, for as long as possible.

That you can still do a few things that you want to do. […] As long as you can do it, you should keep doing it. It also clears your head, doesn’t it. […] [Focus group 2]

While memory problems impeded several activities (e.g., driving a car), many activities were still undertaken, such as cycling, taking a walk, reading, watching television, and pursuing a hobby. These activities were described in various ways, referring to what people gained from engaging in such activities: *feeling useful* and *relaxing*.

##### Feeling useful

It was important for many participants to help others, for instance, by involving others in conversations and activities, or helping neighbors with practical matters.

I’ll go to one lady now, […] She is visually impaired and can hardly hear anything either. She hardly ever left the house. Then they asked if I would like to go for a walk alone with [her], I do that in the afternoon, then I go for a walk with her in the wheelchair for an hour. [Jakob]

Faith was described as a reason to help others, and for some, helping others was the main reason for attending a day care facility. Keeping the household running (grocery shopping, running errands, cleaning) was an important way of helping a partner, or just as “a normal thing to do.”

So cleaning, ironing shirts, vacuuming, cleaning things up. Then when I’ve picked up [my wife], [preparing] dinner. […] Certain things, that the potatoes are ready. [Albert]

Finally, useful activities to train body and mind were described, such as learning new things, and taking walks or exercising.

##### Relaxing

Participants engaged in several individual recreational activities to relax, such as watching television, listening to music, and gardening. Other social recreational activities were also mentioned, such as playing card games, billiards, or darting. Finally, several recreational activities were undertaken both alone and with others, in order to get out and see more of the area, other cities, and nature:

And every day I cycle a bit on my bike here in the area and so on. I also go for short walks. I just like it. And we now live here in the city center, well, in the city anyway. That is also interesting. [Gerrit]

And furthermore: I puzzle a lot. Yes. Yes. Yes. Jigsaw puzzles I mean, huh? Nice. Yes. [Focus group 1]

No, no, I like it for the watering can, watering the vegetable garden a bit, but otherwise I like it. And the, growing things too. we have a few fruit trees here in the garden. [Wouter]

#### Subtheme: engaging with others

A second way to live a fulfilling life was by engaging with others. Various good relationships were mentioned: family, especially (grand)children and partners, was most important for some. Additionally, old friends, good neighbors who became friends, and other people with dementia who visited the same day care facility provided good relationships. However, merely being around people, regardless of the level of intimacy of the relationship, was also an important way of having contact with others. Engaging with others was either initiated by the person with dementia or by other people, depending on the situation and persons involved. Three categories could be distinguished regarding what people gained from these contacts, ascending in intensity: *interacting*, *sharing*, and *relying on others*.

##### Interacting with others

Engaging with others could be very low threshold, by simply going outside and being able to see or greet people, having some friendly eye contact, or a short chat. Going to a campsite could be a way to see other people and have such chats. Drinking coffee was often used as a synonym for chatting:

And then you sometimes feel lonely and if that is the case, I grab my coat and go for a nice walk. […] And then sometimes I run into someone who says: “Ah, would you like to join us for a cup of coffee or a cup of tea?” [Maria]

Chatting with peers in a day care facility was also described, although it could be a challenge to include others in conversation:

Look, you have the idea that you can pick people with whom you can possibly really talk, but well, they leave, eh, so you can no longer talk to them. […] But yes, I am someone who occasionally talks to everyone everywhere and what they have done in life and what hobbies they have etc. [Cornelis].

##### Sharing with others

A more intense level of social contact was having people around who make sure they are okay and who give them attention. For instance, being able to share things with other people with dementia in a day care facility, joking around, and making others feel welcome in the group.

Here the [day care facility] for example. Awesome. […] Well, the whole entourage. The attention they give to the people there. I enjoy that very much. [Eduard]

People are open to questions and answers, in other words, we share a lot with each other. […] Then I also had a conversation in the group, then I said why do I come here? Well to listen, to ask questions, but also to share. And I have to say, those two factors, that works great. [Willem]

##### Relying on others

Having a social network of family, friends, neighbors, and/or formal caregivers on whom they could rely when needed was important, for practical matters, or to arrange social contacts and daily activities.

Yes, but look, the person who holds sway here is my wife. So, she comes up with ideas about what we are going to do and then I can say no or say yes, but it is always fun. [Cornelis]

Well, what comes to mind, the way I approach life, meaning how I can especially rely on the people around me, especially the children, which is very important to me. […]

[about neighbors] Well that is…they want to help once in a while, insofar as I can’t get the TV adjusted properly then that doesn’t work anymore […] and in such cases they will help you. [Dirk]

What I notice is that I have so many more people around me than I had before. I mean I did have a lot of people […] But now I notice personally that there are an incredible number of people who are willing to do something for you and yes, that is also so beautiful to see. [Elisabeth]

### Theme “Having a Positive Attitude Toward Life”

The second main way to realize well-being was by having a positive attitude toward life. Although a few participants mentioned that their condition made them having a slightly less positive attitude toward life, mostly, people described how they tried to view their lives and what happens to them through a positive lens, both in terms of *appreciating the good things in life* and *coping with difficulties in a positive way.* This was addressed in all interviews except one, and both focus groups.

#### Subtheme: appreciating the good things in life

Participants appreciated the good things in life. This refers to the positive and grateful way people look at their life. Three categories could be distinguished that refer to *appreciating current life*, *the lived life*, and *oneself*.

##### Appreciating current life

Current life was appreciated by various people, in terms of things generally going well without significant problems. Faith was described as one reason to remain positive. Children and grandchildren were often important sources of enjoyment and appreciation, particularly, keeping track of and sympathizing with how they were doing.

I have everything I need. The children come and bring food, the nurse comes, the cleaning lady … so. [Pieter]

I’m still doing okay, really. Everything has become different and a little more difficult. But what I have, it’s still good. [Albert]

Well, in terms of my children who are happily married and my children’s children who are too, those are nice children and yes, everything is going well. [Cornelis]

I wanted to say that [family] is the most important thing. [Focus group 1]

The good living situation in the Netherlands, and having a nice home were appreciated, as were various small daily things, such as enjoying good weather and nature

Enjoying, for one, the fact that I exist. And yes, like the sun, and music. That is for me. Then I’m always so happy. […] I had a nice coffee, I slept well. And you should also do something about the housework, too. And I just know, this is going to be a fun day. [Hendrik]

Things are going really well, right? The beautiful weather, what more could you want. [Jan]

##### Appreciating the lived life

Some participants looked back and appreciated the life they had lived, for instance, the joys of their wedding, and their children being born. The lived life was also appreciated more in general terms, or in terms of the lessons they learned.

I’ve already had a full life. [Jan]

I’ve just been through a lot of miserable things, but I’ve also just been so lucky. […] So, but those sound like serious things, but if I hadn’t experienced this then, how would I have found out all about it? Do you understand? So, I’m just thankful that I came across things that helped me. [Elisabeth]

##### Appreciating oneself

A positive attitude toward oneself was also mentioned a few times, by describing confidence in one’s own abilities, and being proud of oneself.

Yes, and being happy with yourself. That is also very important. […] Addressing things that aren’t true and that I’m sick. There is something in my head… And whatnot. […] but then you’re preoccupied with yourself and you just shouldn’t do that. […] You have to be satisfied with yourself. […] Well, [my daughter] has often said to me, “You have to be yourself.” So yes. [Maria]

#### Subtheme: coping with difficulties in a positive way

A positive attitude toward life was also realized by coping with difficulties in a positive way. People faced a variety of challenges, such as having dementia and related problems, but also not having children, the illness of a family member, or the coronavirus disease 2019 pandemic restrictions. Three categories could be distinguished in coping with such difficulties: *accepting difficulties as part of life*, *not dwelling on difficulties*, and *actively addressing difficulties.*

##### Accepting difficulties as being part of life

Several participants described that everyone has to deal with negative things, which is part of life. Dementia was “just” something they had to deal with, for instance, by being at peace with the situation, and accepting instead of fighting not being able to do everything anymore. It varied from time to time how well this went.

It’s all good. At least, I mean, what my trouble is, is now. But I suppose that it’s part of the package, so we just have to accept that. And they say, you can live to be a hundred with it, that is something to hold on to. [Willem]

Yes, then you come across things that you could do before and can no longer do, you just accept that. It’s a matter of acceptance. [Dirk]

[about getting angry because of dementia]: But I realized that I’m not going to do that, that doesn’t work. I understand very well why it happens, but if everyone starts to do that when they get dementia, that would be quite something. I feel sorry for all those people around them. That’s not how life works. Yes, [you have a] responsibility towards others too, whether you have dementia or not. [Elisabeth]

##### Not dwelling on difficulties

It was generally important not to worry, nor complain too much, and to try not to make things bigger than they are. Some participants actually felt lucky, because things could be worse. Furthermore, when something was not going well, or impossible, it was important to try and find alternatives.

[about not being able to travel anymore] Yes. Life offers enough to do, then I will just not go abroad. [Berend]

Look, one has Parkinson’s, the other has Alzheimer’s. Parkinson’s is very bad because then you start to tremble and whatnot. And Alzheimer’s, yes, that is also slightly worse. I think I’m lucky in that regard, that I have vascular dementia. [Cornelis]

When things really aren’t going well, then I do get a little mad at myself. But you know what to do, if something doesn’t work, go do something else. […] Then I’ll do something else. But then I will continue again, then I think ‘maybe it will work now’. And then it usually does. [Hendrik]

##### Actively addressing difficulties

Finally, some difficulties were actively addressed, for instance, by focusing on the things that can be changed, and by trying to make the most of the situation. People described dealing with dementia by relying on family to create some structure, being able to laugh about the problems, and being open about dementia. Leaving things as organized as possible for family was also important, by writing their will, putting the finances in order, and tidying up the house.

Work on what you can do something about. [Focus group 2]

[…] back then with my mother. Well, then it was secret, you didn’t talk about that. […] That’s the difference between then and now. That is. The idea that I told my family […] And for example I told the neighbors. And my sister-in-law lives here in [the city]. […] They know. [Anna]

Yeah, but hey, look, I’d rather face reality. If that’s me, well then I’m not confronted with it, but my family is. With bad things: ‘If only Dad had arranged it then, if only they had done it.’ So I try to prevent that as much as possible. [Willem]

## Discussion

The current study examined how people living with early dementia realize their well-being, without treating dementia as a central concern in the conversations or methods. We aimed to include people who are able to realize high levels of well-being. We found two main themes in how participants attained well-being: (1) living a fulfilling life and (2) having a positive attitude toward life.

The first main way to realize well-being was to *live a fulfilling life*, by engaging in activities to feel useful and to relax, and by engaging with others to interact and share with, and rely on them. One specific activity can thus serve multiple purposes, for instance, taking a walk can train the body, get one out of the house, but also help to meet others at the same time. Our results are in line with previous literature, showing that people with dementia engage in meaningful activities to feel useful, to have personal time and rest, and also to engage with others (Han et al., 2016). More specifically, the ascending intensity of what people gained from these contacts was also found in a previous systematic review: to be with others, participating in social activities, and having meaningful relationships (Han et al., 2016). A day care facility supported living a fulfilling life for many participants in our study, by providing activities and interaction with peers, in line with previous literature (Soderhamn et al., 2014).

The second main way to realize well-being was to have a *positive attitude toward life*, by appreciating the good things in life, and coping with difficulties in a positive way. The appreciative attitude fits the concept of gratitude: the appreciation of the positive things in life and the goodness of others (McCullough et al., 2002). Previous literature has covered the gratitude that people with dementia have about the care they receive (e.g., Goossens et al., 2020). However, our results showed a broader range of topics to be appreciative of: current life, the lived life, and oneself, signifying a need for more comprehensive research of gratitude experience by people living with dementia (McGee et al., 2017). Furthermore, our research demonstrates various strategies for dealing with dementia and other difficulties. Accepting difficulties as part of life, not dwelling on the negative, and actively addressing difficulties are in line with the emotion-focused and problem-focused strategies in persons with dementia described in previous literature (Bjørkløf et al., 2019). In the current study, these positive coping skills extended beyond dementia to other challenges such as not having children or illness of a family member (Wolverson et al., 2016). With this, our results underline a breadth of well-being factors that future interventions in dementia care could target, reaching beyond the dementia condition.

Still, not making dementia the central focus in our study did not lead to substantially different results than previous research. The current themes and categories are comparable to a recent study on the everyday lives of Dutch people with dementia (Huizenga et al., 2023). When comparing our results with the recent conceptualized framework of living well with dementia (Clarke et al., 2020), our subthemes of engaging in activities and engaging with others show great similarities with their domains of *keeping going and being active*, and *good relationships.* Appreciating the good things in life and coping with difficulties in a positive way are in line with their *positive sense of self*, *life having meaning*, and *feeling positive.*

On the other hand, all these (sub)themes appear to reflect rather universal ways of realizing well-being. There are several universal theories of well-being, for example, the theory of Psychological Well-Being (PWB; Ryff, 1989), Self-Determination Theory (SDT; Ryan & Deci, 2017), the PERMA model (Seligman, 2011), or Social Production Functions (SPF) theory (Lindenberg, 2013) and related Self-Management Well-being (SMW) theory (Steverink et al., 2005). These theories include factors related to *engaging in activities* in some form, describing the need for competence and autonomy (SDT, PWB), purpose in life and personal growth (PWB), meaning, engagement, and accomplishment (PERMA), and stimulation and status (SPF). All these theories also include aspects of *engaging with others*, in terms of the need for relatedness (PWB, SDT), having positive relationships (PERMA), or the need for personal affection and behavioral confirmation (SPF). And finally, aspects of the *positive attitude toward life* are also included in these theories, although to a lesser extent: self-acceptance and self-efficacy beliefs (PWB, SMW), and a positive frame of mind concerning the future (SMW). This supports the argument that living a good life does not become fundamentally different when living with dementia (Gerritsen, 2021), and justifies the use of universal (western) theories of well-being in research with people with dementia.

### Strengths and Limitations

The current study is one of the first to examine how people with dementia describe realizing their well-being, without dementia being treated as a central concern by the researchers, and in a non-English speaking country. We generally succeeded in our aim, as we did not include dementia in the interview questions, nor did we use a dementia-specific framework to analyze the data, nor included dementia tests before the interviews. However, participants knew we were aware of their condition, because we recruited via day care facilities and dementia case managers, and mentioned memory problems in the recruitment message. Importantly, most participants themselves were aware of their condition, which may also explain why the topic of dementia came up spontaneously during most interviews (e.g., how they tried to cope with dementia in a positive way). Future studies may want to explore experiences between people who are and who are not aware of their memory problems.

Further strengths of our study include examining well-being in terms of satisfaction and enjoyment, to cover both the cognitive–evaluative and emotional domains of the widely adopted conceptualization of subjective well-being (Diener, 1999). Additionally, to ensure credibility, we used method triangulation, by combining interviews with focus groups, and were able to include a relatively large sample. The rather smooth recruitment process also indicates the willingness and ability of people with dementia to talk about this topic and thereby contribute to such research. To increase the likelihood of participation, we further aspired to create a safe environment for participation (e.g., recruitment through trusted people, a familiar environment, allowing presence of informal caregivers). We do not, however, have insight into the numbers and reasons for participant refusals, or how the nonrespondents differed from our study sample, and therefore do not know to what extent our results are biased by this selection.

An important limitation is that while we aimed to include people with high well-being, a few participants rated their well-being as low to moderate, and generally spoke in more negative terms than other participants. However, these people felt the recruitment message was also aimed at them, and well-being levels are known to vary across time (Kolanowski et al., 2007). Furthermore, the main themes were present in each conversation, indicating important ways of realizing well-being for everyone. For these reasons, we did not exclude participants based on their well-being ratings. Nevertheless, future studies may wish to compare descriptions of people with high versus low well-being, to examine whether they differ in the way they try to realize their well-being, or in the extent to which they are successful in doing so.

Furthermore, there are some limitations to the potential impact of our rather homogenous sample on the results, as the sample mainly consisted of Dutch White men, with early to moderate dementia, and with a partner and children. The Dutch healthcare system is known for its high dementia care standard (Huijsman et al., 2020). Indeed, all participants visited a day care facility or had a case manager (Reinhoudt-den Boer et al., 2022). Our sample consisted of 86% men, while only about 36% of Dutch people aged 65+ who are living with dementia are male (Nivel, 2021). It remains unclear whether men felt more often that the recruitment message was aimed at them, or whether contact persons attributed high well-being more to their male than their female clients. People of minority ethnic groups were underrepresented, which is in line with them experiencing more difficulty with finding their way to dementia care facilities (Mukadam et al., 2011). Importantly, the experience of life with dementia may vary with social location (Hulko, 2009). For instance, the coping resource of family support (Bjørkløf et al., 2019) may have affected the way our participants described their positive coping strategies. In this way, the homogeneous sociodemographic characteristics of the sample may have limited the universality of the well-being descriptions in our study. The impact of intersectionality and the transferability of our results to people in other (non-Western) countries, who do not visit a day care facility and those who are experiencing more advanced levels of dementia, as well as potential age differences, should be further examined.

## Conclusion

This study has shown how people living with dementia are able to realize their well-being. People living with dementia describe that a good life includes living a fulfilling life and having a positive attitude toward life, by means of engaging in activities, engaging with others, appreciating the good things in life, and coping with difficulties in a positive way. Dementia appears to be seen as one such difficulty. The results reflect universal ways of achieving well-being. This supports the use of universal models of well-being for people with dementia, and provides a potential framework for developing well-being interventions.

## Supplementary Material

gnae077_suppl_Supplementary_Materials

## Data Availability

The interview guide is available in the Supplementary Material. Data are not available to other researchers, due to ethical considerations of true anonymization of the full transcriptions. The study was not preregistered.
