# Supplementary material for: Learning From People With Dementia What Works Well for Well-Being: Interviews and Focus Groups
Source: Gerontologist. 2024 Jul 2;64(9):gnae077. doi: 10.1093/geront/gnae077 (PMC11308173; doi:10.1093/geront/gnae077)

**Supplementary Material**

**Interview guide**

‘What works well for well-being’

Questionnaire and Topic guide

**Research**

We are curious about how people can enjoy and be satisfied with life, so that we can learn from it and help others too. That's what I'd like to talk to you about today.

We use your answers to help others and to write an article.

I would like to record this conversation with this device so that I can listen to it later. This is registered anonymously, so without your name. Is that okay?

I also take some notes during our conversation so that I can keep a good record of what we discussed.

The investigation takes about an hour, depending on what you want to say.

I am especially curious about your experience.

There are no right or wrong answers, it's really about what you think.

Feel free to ask a question during the conversation if something is not clear.

Do you have a question about what that means?

**Consent Form**

Before we begin, I would like to ask you to sign the consent form.

By that you say that…. [*go through the form*]

**Start**

Then I would like to start the conversation now, for that I turn on the recorder.

**[Name date, place, ppnr]**

**Descriptives**

**1. What is your sex?**

- Male
- Female

**2. What is your age?**

______ year

**3. How is your health in general?**

- Excellent
- Very good
- Good
- Moderate
- Bad

**4. What is the highest level of education you have completed (with a diploma)?**

**5. What was your (longest) occupation?**

**6.** **What is your marital status?**

- Married
- Not married, no partner
- Long-term cohabitation, unmarried
- Divorced
- Widow / widower / partner deceased

**7. Do you have children**

- no
- yes

**8.** **What is your living situation?**

- Independent, alone
- Independently, with others (partner, children, etc.)
- Other, namely: _________________________

**Interview Questions**

When you look at life in general,

**1. How satisfied are you with your life?**

*And if you had to give it a rating? [report mark to VAS-scale]*

***What comes to mind when you think about being satisfied with life?***

**2. Do you enjoy things in life?**

*And if you had to give it a rating? [report mark to VAS-scale]*

***What comes to mind when you think about enjoying life?***

**3. What do you have to do to have a positive outlook on life?**

**4. Are you having an enjoyable day today**?

- What makes this an enjoyable/nice day?

1. **Do you have to do your best to make the most of the day?**
   - How do you do that?

- When is that not possible?

Many people are looking for how to be content and enjoy life.

**6. What would you recommend others to feel good? // have a positive outlook on life?**

- What can they do for that?

**Ending**

I have come to the end of the conversation. Is there anything else you would like to add?

Thank you very much for allowing me to interview you, you helped us a lot. We can also learn how to help others to experience more pleasure and satisfaction.

How did you like talking about this with me?

Do you have further questions?

- Would you like to receive further information about the results of the survey?

**[recorder off]**

**[For (informal) caregiver]**

**9. What form of dementia does this person have (if known)?**

- Alzheimer's disease
- Frontotemporal dementia
- Vascular dementia
- Mixed Vascular/Alzheimer's
- Alcohol Dementia
- Dementia by Lewy Bodies
- Parkinson's Disease Dementia
- Not otherwise specified
- I don't know
- Other, namely: _________________________

**10. When was this diagnosis made?**

- Month: ___________ Year: __________

**11. What stage of dementia do you think your loved one is in?**

- An early stage (mild dementia)
- An intermediate stage (moderate dementia)
- Late stage (severe dementia)

**Satisfaction with life**

**How satisfied are you with your life?**

Can you give a score to that:

**Fully**

**satisfied**

**Fully**

**dissatisfied**


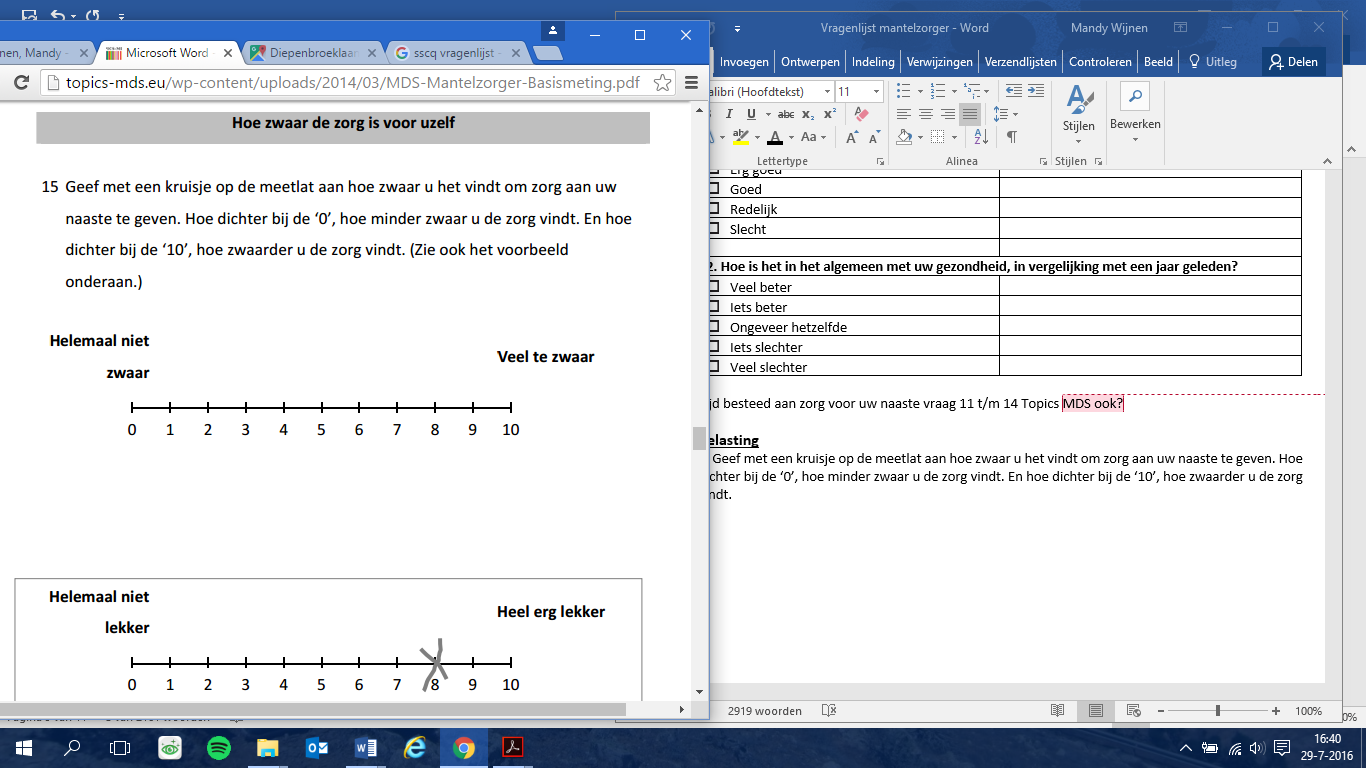


**Enjoying things in life**

**Do you enjoy things in life?**

Can you give a score to that:

**Never enjoying**

**Always enjoying**


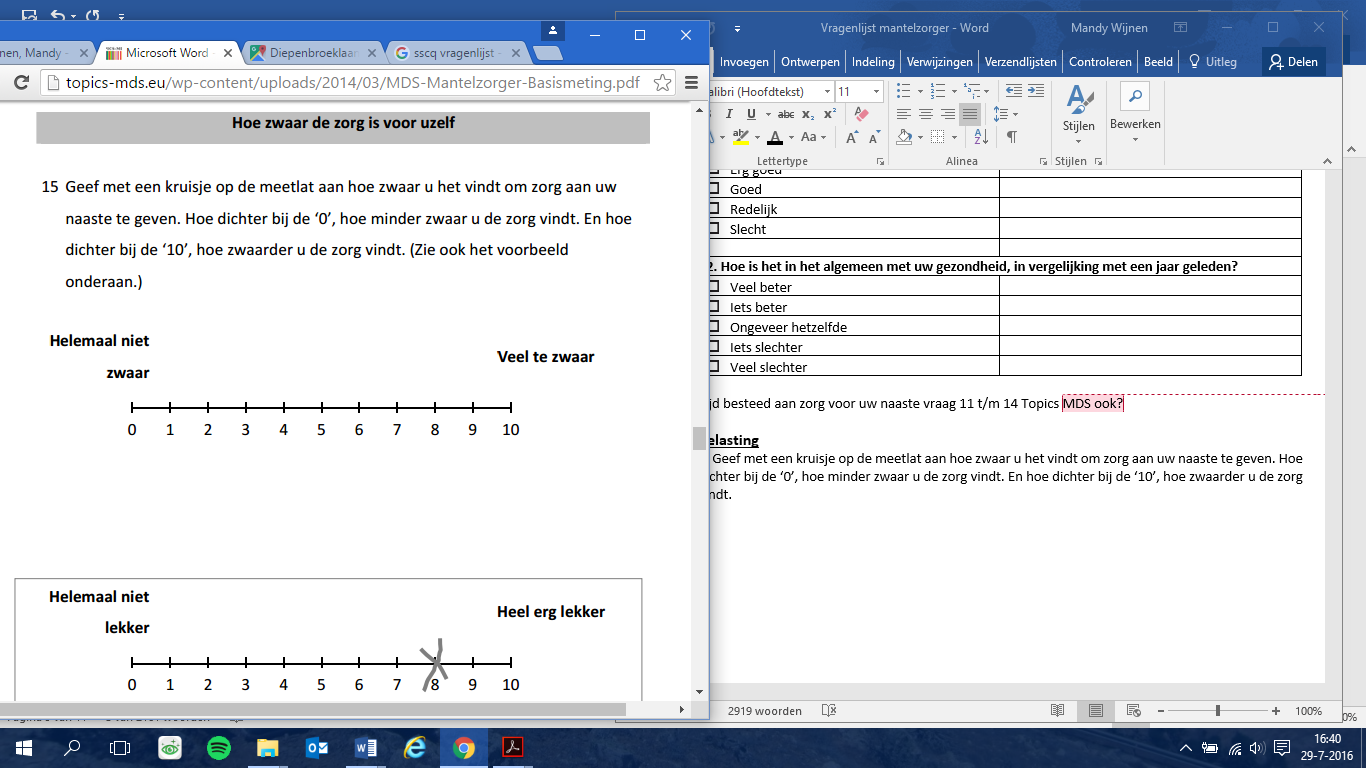

Supplement: gnae077_suppl_Supplementary_Materials [file gnae077_suppl_supplementary_materials.docx]
